# Oral microbial and metabolic alterations in acute exacerbations of COPD across fungal status

**DOI:** 10.3389/fcimb.2026.1843568

**Published:** 2026-10-15

**Authors:** Yanmei Feng, Fengjie Tang, Qihuan Ma, Zhi Ao, Qian Xiong, Wei Qin, Youjing Yang, Shasha Tao, Rui Guo

**Affiliations:** 1 Department of Respiratory Medicine, Chongqing Key Laboratory of Emergency Medicine, Chongqing Emergency Medical Center, Chongqing University Central Hospital, Chongqing, China; 2 Department of Respiratory and Critical Care Medicine, The First Affiliated Hospital of Chongqing Medical University, Chongqing, China; 3 Chongqing Key Laboratory of Emergency Medicine, Chongqing Emergency Medical Center, Chongqing University Central Hospital, Chongqing, China; 4 Department of Emergency Medicine, The First Affiliated Hospital of Chongqing Medical University, Chongqing, China

**Keywords:** AECOPD, *Aspergillus*, *Candida*, oral microbiome, untargeted metabolomics

## Abstract

**Background:**

Oral dysbiosis has been associated with inflammatory processes in acute exacerbations of chronic obstructive pulmonary disease (AECOPD). However, whether Aspergillus-associated or Candida-positive status is associated with distinct oral multi-omics profiles, and whether these profiles change following treatment, remains unclear.

**Methods:**

In this prospective cohort study (August 2024 to July 2025), 18 AECOPD patients were enrolled and stratified into non-fungal exacerbators (n=6), *Aspergillus*-associated AECOPD (n=6), and *Candida*-positive AECOPD (n=6). Oral samples were collected before treatment and, where available, after treatment. The primary analyses compared pretreatment microbial and metabolic profiles across fungal-status groups, whereas longitudinal pre/post-treatment analyses were secondary and exploratory and were performed only in groups with sufficient paired samples.

**Results:**

Baseline characteristics were comparable across groups. At pretreatment, Aspergillus-associated AECOPD showed significantly reduced α diversity and distinct microbial community separation from non-fungal exacerbators (Bray–Curtis R = 0.378, P = 0.009), whereas Candida-positive AECOPD showed no significant global shift. At the genus level, exploratory LEfSe analysis identified distinct subgroup-associated candidate taxonomic signatures. PICRUSt2-based predicted functional profiles were broadly similar across groups. Metabolomic profiling showed the most pronounced baseline differences in the Aspergillus-associated group, with 11 upregulated and 90 downregulated metabolites versus the non-fungal group. Differential metabolites in this baseline comparison were predominantly mapped to sphingolipid metabolism, purine metabolism, one-carbon metabolism, and cAMP/cGMP signaling pathways. In secondary exploratory longitudinal analyses, metabolic shifts were observed in the non-fungal group (4 upregulated and 78 downregulated metabolites) and the Candida-positive group (14 upregulated and 8 downregulated metabolites). No formal longitudinal analysis was performed in the Aspergillus-associated group because only two post-treatment samples were available. O2PLS identified shared cross-omics variation distinguishing pretreatment Aspergillus-associated from non-fungal AECOPD, with several microbial and metabolite features contributing to the shared latent component.

**Conclusion:**

Compared with non-fungal and *Candida*-positive AECOPD, *Aspergillus*-associated AECOPD showed the most pronounced pretreatment oral microbial dysbiosis and metabolic alterations. These findings support the presence of a distinct oral microbial–metabolic phenotype associated with *Aspergillus* status and provide candidate signatures for future validation in larger longitudinal cohorts.

**Clinical Trial Registration:**

https://www.chictr.org.cn/hvshowproject.html?id=250288&v=1.0, identifier ChiCTR2400083090.

## Introduction

Acute exacerbations of chronic obstructive pulmonary disease (AECOPD) are major determinants of disease progression, healthcare burden, and mortality. Although bacterial infections are well-established triggers, emerging evidence highlights AECOPD as a biologically heterogeneous syndrome in which distinct infectious etiologies elicit divergent inflammatory responses and clinical trajectories (Xie et al., 2025). Fungal pathogens, particularly *Aspergillus* and *Candida*, have recently gained recognition as underappreciated contributors to disease burden, especially in patients exposed to corticosteroids, broad-spectrum antibiotics, or recurrent hospitalizations (Luo et al., 2025; Tiew et al., 2021). In China, pathogenic fungal infections have become an increasing public health concern, while their often-nonspecific clinical presentations may complicate timely recognition, particularly in patients with underlying respiratory diseases (Beibei et al., 2026). However, whether different fungal status is associated with distinct host-microbial interactions beyond the lower airways remains poorly understood.

The oral cavity serves as a critical ecological interface linking the external environment, respiratory tract, and systemic immunity. Oral microorganisms are frequently aspirated into the lower airways, and the metabolites can modulate pulmonary inflammation and immune response (Gaeckle et al., 2020; Wu et al., 2021). In COPD, oral dysbiosis has been associated with systemic inflammation, altered immune responses, and increased exacerbation risk (Wen et al., 2022). Nevertheless, most airway microbiome studies in COPD have focused on sputum or bronchoalveolar lavage fluid (BALF), the oral microbiome has received comparatively little attention despite its accessibility and biological relevance.

Fungal colonization and infection may disproportionately affect oral microbial ecosystems because of their distinct ecological niches and metabolic demands (Kashyap et al., 2024; Rozaliyani et al., 2023). *Aspergillus* species are filamentous fungi capable of extensive tissue invasion and potent immune activation, whereas *Candida* species typically behave as commensal yeasts with opportunistic pathogenicity under conditions of immune imbalance. These biological differences suggest that *Aspergillus*-associated and *Candida*-positive states may be associated with distinct oral microbial community patterns in AECOPD, although their relationships with inflammatory and metabolic phenotypes remain to be clarified. More broadly, respiratory microbial dysbiosis in COPD has been associated with inflammatory phenotypes and disease severity (Wang et al., 2023). However, direct comparative data examining pathogen-specific oral microbiome remodeling in COPD remain scarce.

Beyond taxonomic composition, microbial function is increasingly recognized as a key determinant of host-microbe interactions. Microbial- and host-derived metabolites serve as critical mediators linking microbial dysbiosis to immune activation, epithelial integrity, and systemic inflammation. Untargeted metabolomics offers a powerful approach to capture these functional outputs, yet integrated oral microbiome and metabolomic profiling in COPD remain limited. In this prospective cohort study, we combined 16S rRNA gene sequencing with untargeted LC–MS metabolomics to characterize oral microbial and metabolic profiles across non-fungal, *Aspergillus*-associated, and *Candida*-positive AECOPD groups, and to assess treatment-associated changes where longitudinal samples were available.

## Materials and methods

### Study design

From August 2024 to July 2025, a total of 18 patients with AECOPD were prospectively recruited from Chongqing University Central Hospital and classified according to fungal status. The inclusion criteria were as follows: (1) diagnosed with AECOPD according to GOLD criteria (Agusti et al., 2023). (2) age ≥ 40 years.

For patients with fungal-positive findings, fungal status was determined through an integrated assessment of clinical manifestations, respiratory fungal culture, serum fungal biomarkers (GM and/or BDG), molecular testing where available, chest CT findings, and histopathology where available. Persistent or worsening respiratory symptoms despite antibacterial therapy were considered supportive clinical evidence. Fungal isolation from respiratory specimens alone, in the absence of compatible clinical and radiological evidence, was not considered sufficient to establish invasive fungal infection. Patient-level diagnostic evidence is summarized in **Supplementary Table S1**.

The exclusion criteria were as follows: (1) concomitant respiratory diseases (e.g., asthma, pulmonary tuberculosis, bronchiectasis, sleep apnea syndrome, lung cancer, or interstitial lung disease). (2) chronic treatment with oral corticosteroids or immunosuppressive agents. (3) antibiotic use within 4 weeks prior to enrollment.

Antibiotic, inhaled corticosteroid (ICS), and antifungal exposures, including treatment class or agent, dose and duration where applicable, cumulative exposure, and the timing of oral sample collection relative to treatment initiation, were recorded for each participant and are summarized in **Supplementary Table S2**. Antifungal therapy was administered when clinically indicated based on the treating physicians’ integrated clinical assessment. Patients in the *Aspergillus*-associated AECOPD group received voriconazole-based antifungal therapy for 4–6 weeks, with treatment duration individualized according to clinical response, radiological findings, mycological results, and overall clinical assessment. Patients in the *Candida*-positive group received caspofungin and/or fluconazole for 7–14 days based on the treating physicians’ integrated clinical assessment, including microbiological findings and clinical response. Oral samples were collected using standardized kits at two predefined time points: before treatment initiation and at the end of the treatment course. After collection, oral wash samples were centrifuged, and the resulting pellets and supernatants were used for microbial DNA analysis and metabolomic detection, respectively. Aliquots were snap-frozen in liquid nitrogen and stored at -80 °C until analysis. Demographic and clinical characteristics were balanced across groups. All participants provide written informed consent. The study was approved by the Human Ethics Committees of Chongqing University Central Hospital.

### DNA extraction and PCR amplification

DNA was extracted using the FastPure Soil DNA Isolation Kit(Magnetic bead)(MJYH, shanghai, China). Three sample-free extraction blanks were processed alongside the study samples to monitor potential contamination introduced during DNA extraction. DNA concentration, integrity and purity were assessed by 1% agarose gel electrophoresis and NanoDrop2000. The V3-V4 hypervariable region of the bacterial 16S rRNA gene were amplified with primers 338F (5’-ACTCCTACGGGAGGCAGCAG-3’) and 806R (5’-GGACTACHVGGGTWTCTAAT-3’) on a T100 Thermal Cycler PCR thermocycler (BIO-RAD, USA). Three no-template controls, containing PCR reagents without template DNA, were included to monitor potential contamination during amplification. The extraction blanks and no-template PCR controls were examined by 2% agarose gel electrophoresis, and none showed detectable amplification. Because no amplicons were detected, these negative controls were not advanced to purification, library preparation, or sequencing. No separate positive control or mock-community standard was included. PCR products were examined by 2% agarose gel electrophoresis to verify the presence and approximate size of the target amplicons, purified using the PCR Clean-Up Kit (YuHua, Shanghai, China) and quantified with Qubit 4.0 (Thermo Fisher Scientific, USA). Purified amplicons were pooled in equimolar concentrations and paired-end sequenced on an Illumina Nextseq2000 platform (Illumina, San Diego,USA) by Majorbio Bio-Pharm Technology Co. Ltd. (Shanghai, China). Raw reads were deposited into the NCBI Sequence Read Archive (SRA) database.

Raw sequences were demultiplexed, quality filtered with fastp (v0.19.6) and merged with FLASH (v1.2.11). Denoising was performed using DADA2 plugin in the QIIME2(v2020.2) to generate amplicon sequence variants (ASVs). To standardize sequencing depth, each sample was rarefied to 20,000 reads, yielding an average Good’s coverage of 97.90%. Alpha diversity was assessed using the Sobs, ACE, Chao, Shannon, Simpson, and Good’s coverage indices. Independent pretreatment comparisons between fungal-status groups were performed using the Wilcoxon rank-sum test. Within-group pre/post-treatment comparisons were restricted to participants with paired samples and were performed using the Wilcoxon signed-rank test. The paired analyses included six patients in the non-fungal AECOPD group and four patients in the *Candida*-positive group. No formal paired analysis was performed in the *Aspergillus*-associated group because only two post-treatment samples were available. Beta diversity was visualized using principal coordinate analysis based on Bray–Curtis dissimilarities. Differences in microbial community composition between groups were evaluated using ANOSIM. Taxonomic assignment was performed using the BLAST consensus classifier in QIIME2 against the SILVA 16S rRNA database (v138). SILVA was selected because it provides a comprehensive, quality-controlled, and widely used reference framework with broad bacterial coverage and standardized taxonomy, facilitating reproducibility and comparison with previous 16S rRNA-based studies. Because the primary taxonomic analyses in this study were conducted mainly at the genus level, species-level classification was not emphasized. Linear discriminant analysis effect size (LEfSe) was used as an exploratory analysis to identify bacterial taxa potentially contributing to differences among groups, from the phylum to genus levels. Taxa meeting a nominal Kruskal–Wallis P value <0.05 and an LDA score >2.0 were retained as candidate discriminative features. No additional false discovery rate correction was applied to the LEfSe analysis; therefore, these findings were interpreted as exploratory rather than confirmatory.

Microbial functional potential was inferred using PICRUSt2 v2.2.0-b based on the ASV representative sequences and abundance table (Douglas et al., 2020). The ASV abundance table rarefied to 20,000 sequences per sample was used as input, and the PICRUSt2 pipeline was run using default settings. Briefly, ASV representative sequences were aligned to the reference alignment using HMMER and placed into the reference phylogenetic tree using EPA-NG and GAPPA. Hidden-state prediction implemented in Castor was used to estimate 16S rRNA gene copy numbers and gene-family copy numbers. ASV abundances were normalized by the predicted 16S rRNA gene copy numbers before sample-level functional profiles were generated. Predicted gene-family abundances were output as KEGG Ortholog (KO) profiles. The KO profiles were subsequently mapped to the KEGG pathway hierarchy and summarized as pathway abundance tables at Levels 1, 2, and 3. No additional user-defined abundance or prevalence filtering or data transformation was applied after PICRUSt2 prediction. For group-level visualization, the predicted functional abundances were summarized as the mean across samples within each group.

### Metabolite extraction and UHPL-MS/MS analysis

A 200 μL aliquot of oral samples was mixed with 800 μL acetonitrile: methanol (1:1, v:v) containing four internal standards (0.02 mg/mL L-2-chlorophenylalanine, etc.). Samples were vortexed for 30 s, sonicated for 30 min at 5 °C, and incubated at -20 °C for 30 min to precipitate proteins. After centrifugation (4 °C, 13000 × g, 15 min), the supernatant was removed. The sample was dried under nitrogen and re-solubilized in 120 µL of acetonitrile: water (1:1, v/v). Following sonication (5 °C, 5 min), and centrifugation (13000 × g, 4 °C for 10 min), the supernatant was transferred to sample vials for LC-MS/MS analysis using the UHPLC-Orbitrap Exploris 240 system (Thermo Fisher Scientific).

A pooled quality-control (QC) sample was prepared by mixing equal-volume aliquots from all study samples. One QC sample was injected after every 5–10 study samples throughout the analytical sequence to monitor analytical stability and reproducibility. All study samples were analyzed within a single analytical batch; therefore, no inter-batch correction was required.

Raw LC-MS data were processed using progenesis QI (Waters Corporation,vMilford, USA) to generate a three-dimensional matrix comprising samples, metabolites, and peak intensities. Peaks corresponding to internal standards, noise, column bleed, and derivatized reagents were removed. Raw metabolomic data were further subjected to preprocessing, including missing-value filtering and recoding, data normalization, QC validation, and data transformation, to reduce technical variation unrelated to the biological comparisons of interest. Normalization was performed in Progenesis QI using its default “Normalise to all compounds” approach. QC reproducibility was evaluated before and after normalization using the relative standard deviation (RSD) distribution of detected metabolic features. At an RSD threshold of 30%, 76.34% of metabolic features in the raw QC data and 86.84% of features in the normalized QC data had RSD values below 30% (**Supplementary Figure S1**). Metabolites were identified using the HMDB (http://www.hmdb.ca/), Metlin (https://metlin.scripps.edu/) and the Majorbio Database (MJDB). PCA, PLS-DA, and OPLS-DA were performed using the R package “ropls” (v1.6.2). PCA was performed after unit-variance scaling, whereas PLS-DA and OPLS-DA were performed after Pareto scaling. The supervised PLS-DA/OPLS-DA models were evaluated using seven-fold cross-validation and 200 permutation tests to assess model robustness and potential overfitting. Candidate differential metabolites were selected using a variable importance in projection (VIP) score ≥1 derived from the OPLS-DA model, or from the PLS-DA model when the OPLS-DA model showed evidence of overfitting, together with a nominal two-sided Student’s t-test P value <0.05. Fold change was used to characterize the direction and magnitude of metabolite differences. No formal multiple-testing correction was applied to the metabolite-wise t-tests; therefore, the identified metabolites were interpreted as exploratory candidate features rather than statistically confirmed differential metabolites. Pathway enrichment analysis was conducted using KEGG.

### Integrated microbiome-metabolomics analysis

Procrustes analysis was used to assess global concordance between the microbiome and metabolomic datasets. O2PLS was subsequently applied to identify shared latent variation and the taxa and metabolites contributing to the cross-omics covariance structure. All data were analyzed with the free online platform of majorbio cloud platform (https://v.majorbio.com/) (Han et al., 2024). O2PLS loadings were interpreted as the contributions of individual microbial and metabolite features to the shared latent components and not as evidence of direct pairwise taxon–metabolite associations.

### Statistical analysis

Continuous variables were expressed as mean ± SD and were compared using one-way ANOVA. Categorical variables were analyzed using the chi-square test. Pairwise comparisons were performed using Student’s t-test or Mann–Whitney U test. Statistical significance was set at p < 0.05. All analyses were conducted using GraphPad Prism 9.0 and R v3.4.4.

## Results

### Participant characteristics

A total of 18 AECOPD patients were included: 6 in the non-fungal group, 6 in the *Aspergillus*-associated group, and 6 in the *Candida*-positive group. Most participants were male, and no significant differences were observed across groups in age, body mass index (BMI), inflammatory markers, or blood gas parameters. Baseline characteristics were well balanced (**Supplementary Table S3**). Post-treatment oral samples were obtained from all patients in the non-fungal AECOPD group (n=6), 4 patients in the *Candida*-positive AECOPD group, and 2 patients in the *Aspergillus*-associated AECOPD group. Detailed patient-level evidence supporting the classification of fungal infection, including respiratory culture, GM/BDG testing, NGS, radiological findings, clinical factors, and histopathology where available, is provided in **Supplementary Table S1**. ICS exposure was comparable across groups, whereas antibiotic duration and cumulative antibiotic exposure differed among groups (P = 0.034 and P = 0.040, respectively), with greater exposure in the *Aspergillus*-associated group (**Supplementary Table S2**). Because of the limited number of post-treatment samples in the *Aspergillus* group, these samples were not included in longitudinal pre/post-treatment comparative analyses.

### Pronounced oral microbiome dysbiosis in *Aspergillus*-associated AECOPD

At pretreatment, none of the evaluated α diversity indices differed significantly between the *Candida*-positive and non-fungal AECOPD groups. In contrast, the *Aspergillus*-associated group showed lower Sobs, ACE, Chao, and Shannon indices and a higher Simpson index than the non-fungal AECOPD group, consistent with reduced microbial richness and diversity (**Figures 1**; **Supplementary Table S4**). Bray–Curtis-based PCoA was used to visualize differences in microbial community composition (**Figure 2**). ANOSIM showed no statistically significant pre/post-treatment differences in the non-fungal AECOPD group (R = 0.2400, P = 0.072) or the *Candida*-positive group (R = 0.2064, P = 0.101). In the pretreatment comparison, however, microbial community composition differed significantly between the *Aspergillus*-associated and non-fungal AECOPD groups (R = 0.3778, P = 0.009).

**Figure 1:**
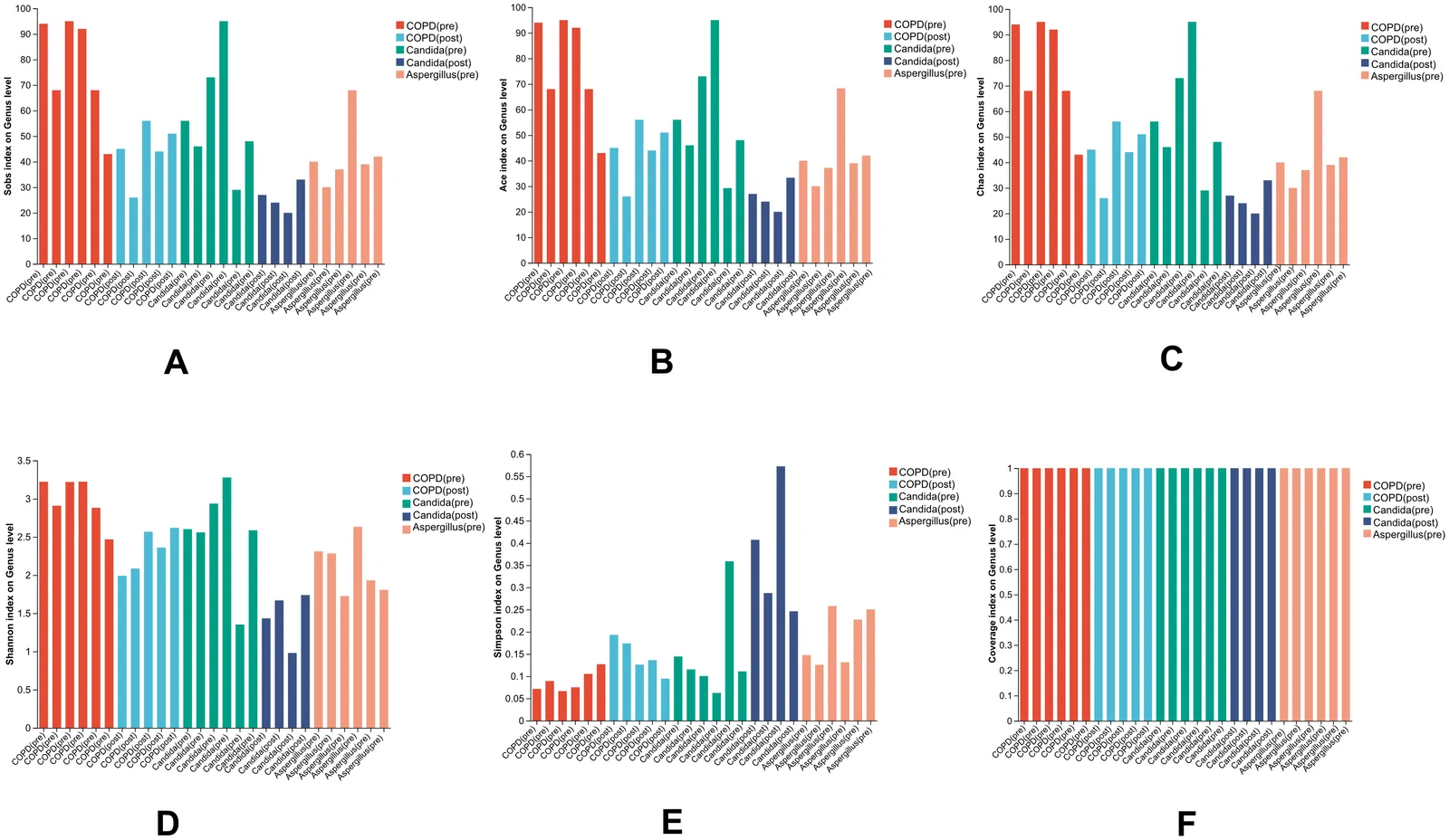
α diversity across COPD subgroups. COPD (pre), non-fungal AECOPD before treatment; COPD (post), non-fungal AECOPD after treatment; Candida(pre), *Candida*-positive AECOPD before antifungal treatment; Candida (post), *Candida*-positive AECOPD after antifungal treatment; Aspergillus (pre), *Aspergillus-*associated AECOPD before antifungal treatment. Pre and post indicate samples collected before treatment initiation and at the end of the treatment course, respectively. The *Aspergillus* post-treatment group included only two available samples and was therefore not included in the longitudinal pre/post-treatment comparative analyses. **(A)** Sobs index. **(B)** Ace index. **(C)** Chao index. **(D)** Shannon index. **(E)** Simpson index. **(F)** Coverage index.

**Figure 2:**
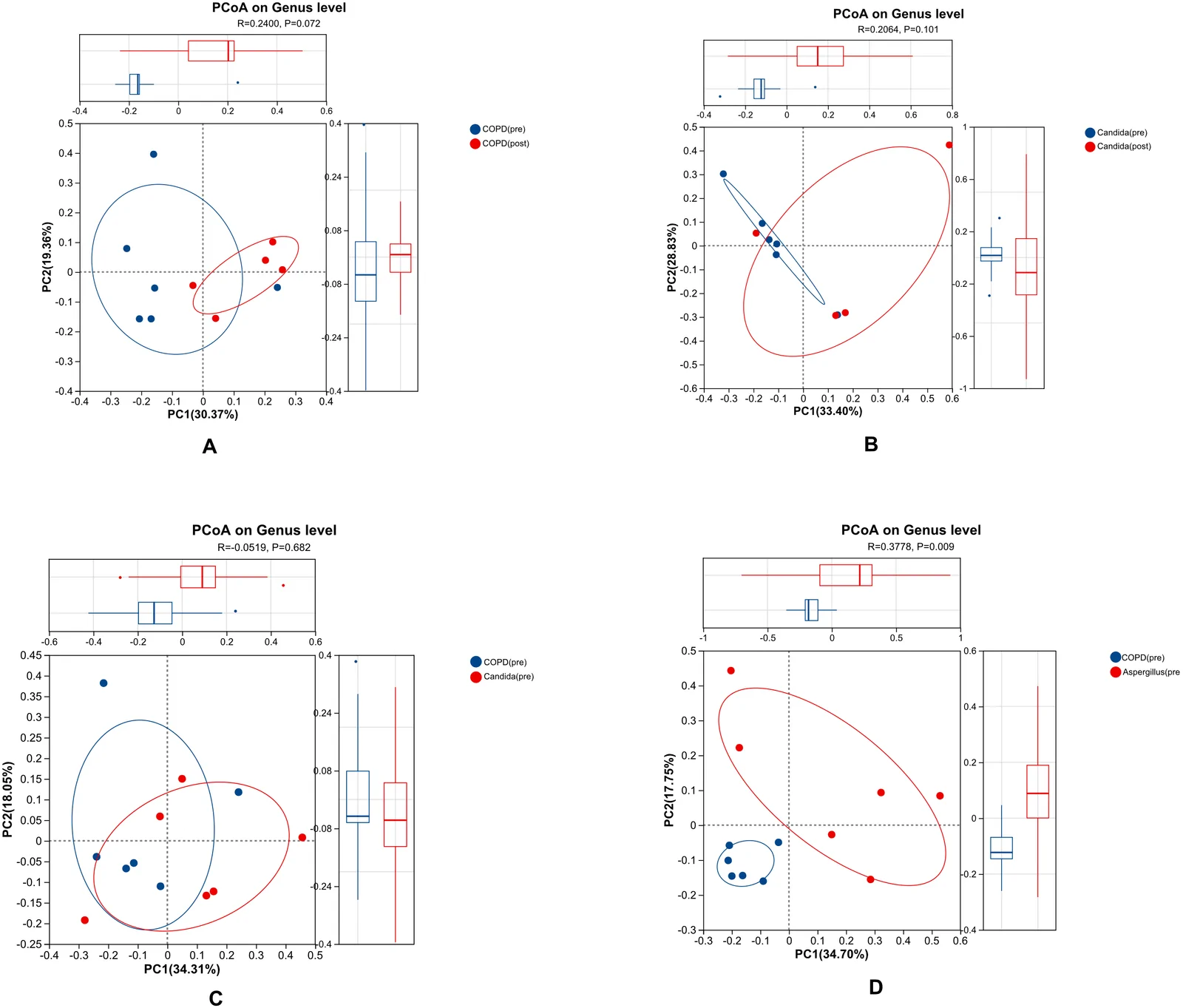
β diversity across COPD subgroups. COPD (pre), non-fungal AECOPD before treatment; COPD (post), non-fungal AECOPD after treatment; Candida (pre), *Candida*-positive AECOPD before antifungal treatment; Candida (post), *Candida*-positive AECOPD after antifungal treatment; Aspergillus (pre), *Aspergillus-*associated AECOPD before antifungal treatment. Pre and post indicate samples collected before treatment initiation and at the end of the treatment course, respectively. Post-treatment samples were available for only two patients in the Aspergillus-associated group; therefore, no formal longitudinal pre/post-treatment comparative analysis was performed for this group. **(A)** COPD (pre) vs COPD (post). **(B)** *Candida* (pre) vs *Candida* (post). **(C)** COPD (pre) vs *Candida* (pre). **(D)** COPD (pre) vs *Aspergillus* (pre). Each point represents one sample.

### Subgroup-specific oral taxonomic signatures

Taxonomic analysis revealed variation in oral microbial composition across the AECOPD subgroups. At the phylum level, the oral microbiome was predominantly composed of Bacillota, Actinomycetota, Pseudomonadota, and Bacteroidota, whereas lower-abundance phyla varied across groups (**Figure 3A**). Fusobacteriota and Patescibacteria showed relatively higher abundances in the COPD-pre group. At the genus level, common oral taxa, including Streptococcus, Rothia, Veillonella, Actinomyces, and Prevotella, were prevalent across groups (**Figure 3B**). Exploratory LEfSe analysis identified subgroup-specific candidate discriminative taxa. Fusobacterium and related Fusobacteriota lineages, together with Patescibacteria- and Saccharimonadia-related taxa, were associated with the COPD-pre group. Neisseria and Porphyromonas were associated with the COPD-post group, whereas Actinomyces and Leptotrichia were associated with the Candida-pre group. Lautropia, Tannerella, *Prevotella pallens*, Capnocytophaga, and Johnsonella were associated with the Aspergillus-pre group (**Figures 3C, D**).

**Figure 3:**
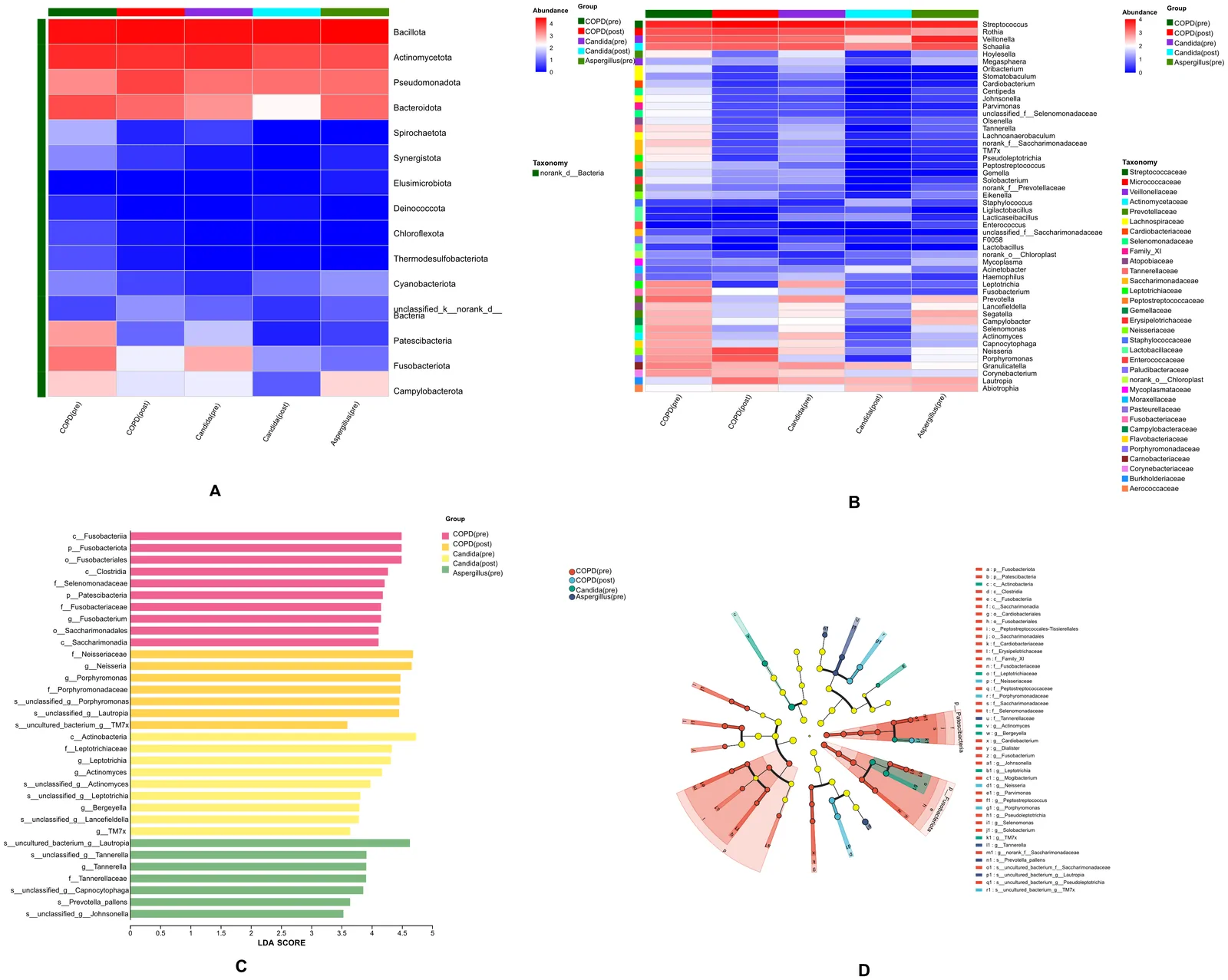
Differential microbial taxa across COPD subgroups. COPD(pre), non-fungal AECOPD before treatment; COPD(post), non-fungal AECOPD after treatment; Candida(pre), *Candida*-positive AECOPD before antifungal treatment; Candida(post), *Candida*-positive AECOPD after antifungal treatment; Aspergillus(pre), *Aspergillus*-associated AECOPD before antifungal treatment. Pre and post indicate samples collected before treatment initiation and at the end of the treatment course, respectively. Post-treatment samples were available for only two patients in the Aspergillus-associated group; therefore, no formal longitudinal pre/post-treatment comparative analysis was performed for this group. **(A)** Heatmap of dominant taxa at the phylum level. **(B)** Heatmap at the genus level. **(C)** LDA score distribution of candidate discriminative taxa identified by exploratory LEfSe analysis. **(D)** Cladogram showing the phylogenetic distribution and subgroup associations of the LEfSe-identified taxa. Panels **(A, B)** show group-level mean relative abundances. The color scale represents relative abundance, with red indicating higher and blue indicating lower abundance. Candidate taxa were selected using a nominal Kruskal–Wallis *P* value <0.05 and an LDA score >2.0. No formal FDR correction was applied; therefore, these taxa should be interpreted as exploratory candidate features.

PICRUSt2-based functional inference showed broadly similar predicted functional profiles across groups (**Figure 4**). Descriptive variation was observed in the predicted abundances of several KEGG pathways and COG functional categories; however, no formal between-group differential testing was performed. Therefore, these patterns were not interpreted as statistically supported pathway enrichment and should be regarded as exploratory.

**Figure 4:**
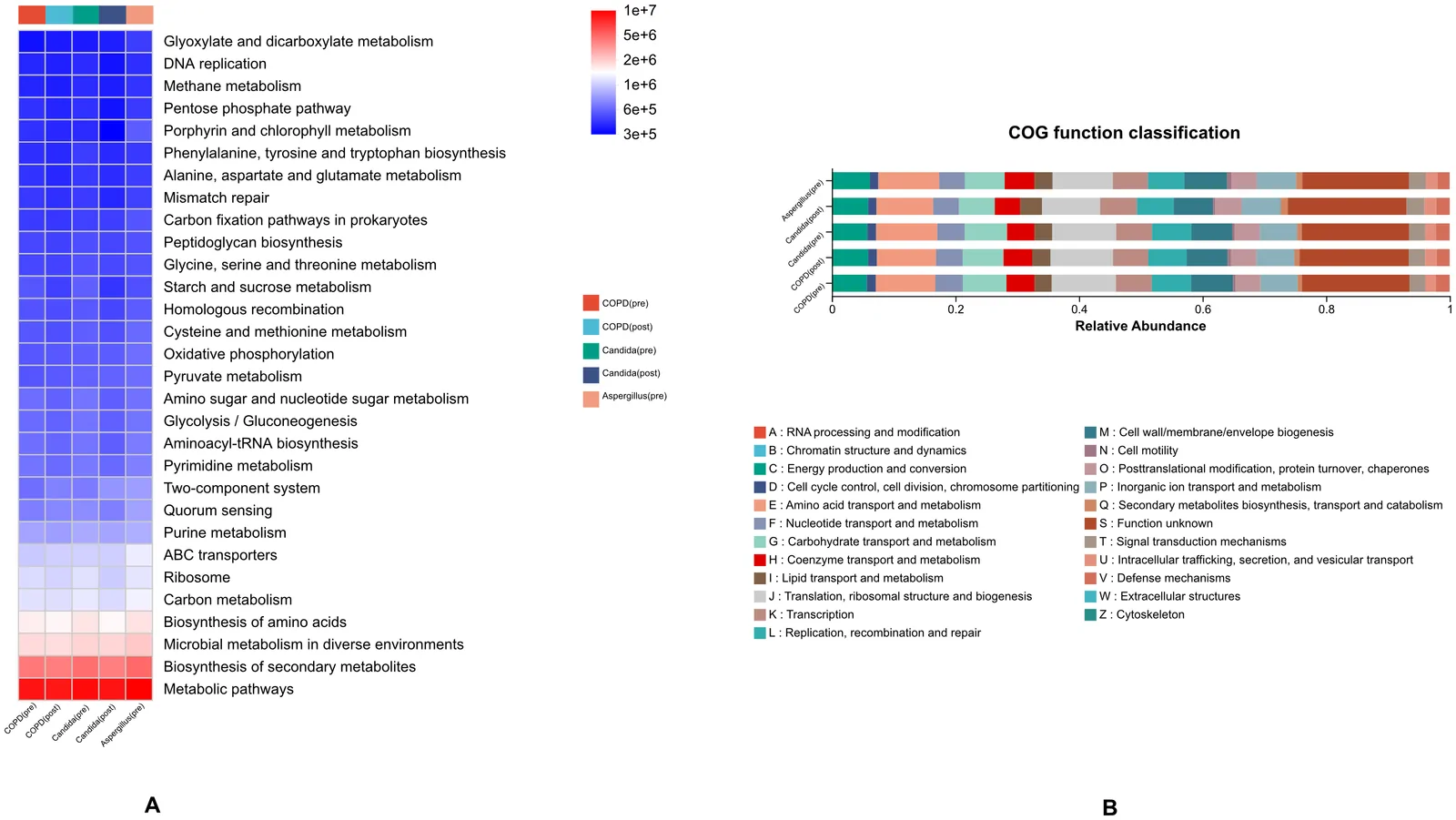
Predicted functional profiles of the oral microbiome. COPD (pre), non-fungal AECOPD before treatment; COPD (post), non-fungal AECOPD after treatment; *Candida* (pre), *Candida*-positive AECOPD before antifungal treatment; *Candida* (post), *Candida*-positive AECOPD after antifungal treatment; and *Aspergillus* (pre), *Aspergillus*-associated AECOPD before antifungal treatment. **(A)** Heatmap showing group-level mean abundances of PICRUSt2-predicted KEGG pathways. **(B)** Relative composition of predicted COG functional categories across groups. The color intensity and relative abundance plots are descriptive and do not indicate statistically significant pathway enrichment. No formal between-group differential enrichment testing was performed; therefore, the displayed profiles are descriptive and exploratory.

### Metabolomic reprogramming in *Aspergillus*-associated AECOPD

Although substantial overlap was observed among the study groups in the PCA model (**Figure 5A**), exploratory PLS-DA suggested separation between the Aspergillus(pre) and COPD(pre) groups (**Figure 5E**). Volcano plots showed treatment-associated metabolic shifts in the non-fungal group, with 4 upregulated and 78 downregulated metabolites (**Figure 6A**), and in the Candida-positive AECOPD group, with 14 upregulated and 8 downregulated metabolites (**Figure 6C**). The Aspergillus-associated AECOPD group displayed the most pronounced baseline metabolic differences compared with the non-fungal group, with 11 upregulated and 90 downregulated metabolites (**Figure 6D**). Hierarchical clustering illustrated variation in the abundance patterns of selected metabolites across the study samples (**Figure 7A**), whereas VIP analysis identified candidate metabolites contributing to the discrimination between the *Aspergillus*(pre) and COPD(pre) groups (**Figure 7E**).

**Figure 5:**
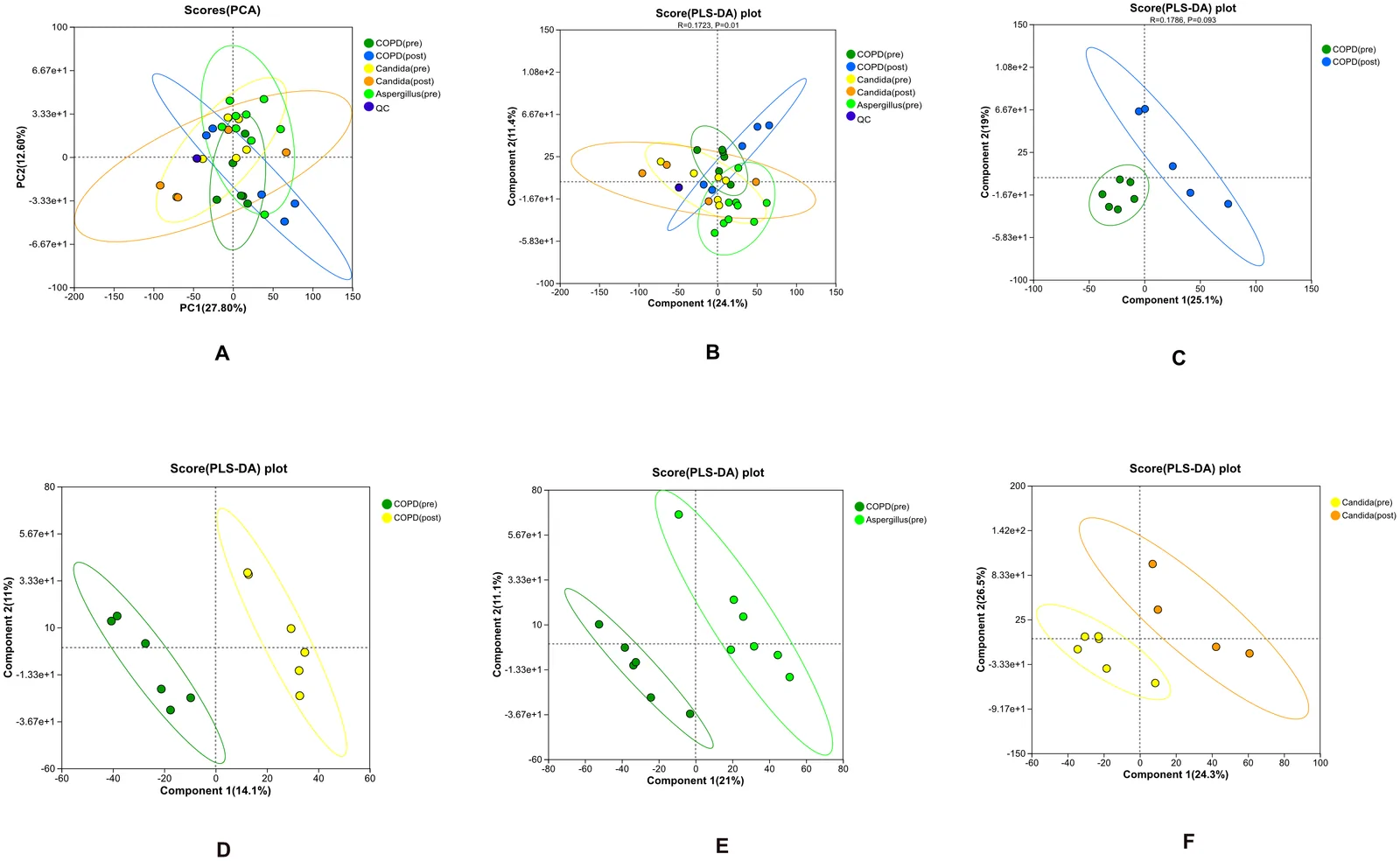
Multivariate metabolomic analysis across COPD subgroups. COPD (pre), non-fungal AECOPD before treatment; COPD (post), non-fungal AECOPD after treatment; Candida(pre), *Candida*-positive AECOPD before antifungal treatment; Candida(post), *Candida*-positive AECOPD after antifungal treatment; Aspergillus(pre), *Aspergillus*-associated AECOPD before antifungal treatment. Pre and post indicate samples collected before treatment initiation and at the end of the treatment course, respectively. Post-treatment samples were available for only two patients in the Aspergillus-associated group; therefore, no formal longitudinal pre/post-treatment comparative analysis was performed for this group. **(A)** PCA score plot including QC samples. **(B)** PLS-DA score plot across all groups. **(C–F)** PLS-DA comparisons between individual COPD subgroups.

**Figure 6:**
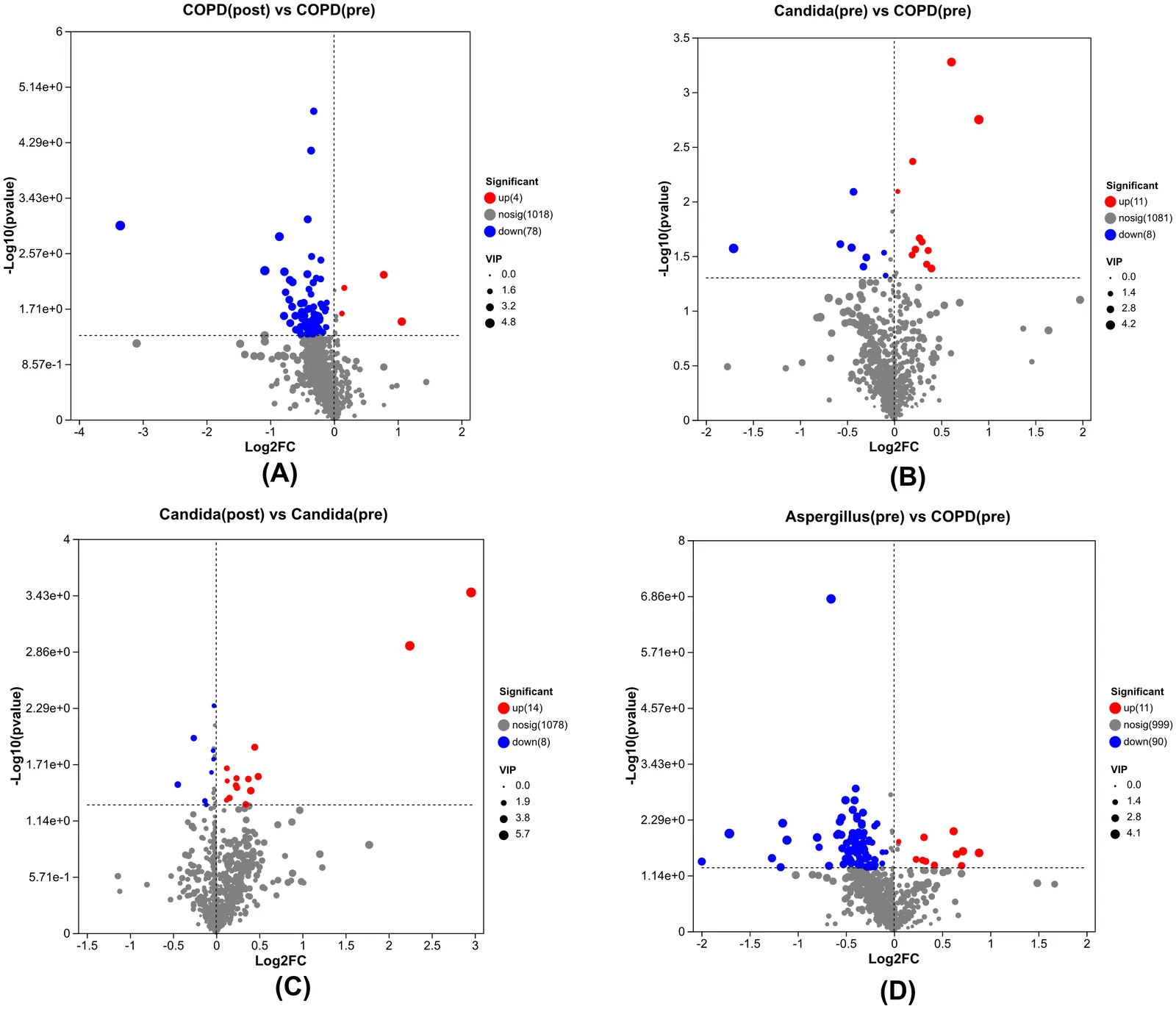
Differential metabolite analysis across COPD subgroups. **(A)** COPD (post) versus COPD (pre). **(B)** *Candida* (pre) versus COPD (pre). **(C)** *Candida* (post) versus *Candida* (pre). **(D)** *Aspergillus* (pre) versus COPD (pre). COPD (pre), non-fungal AECOPD before treatment; COPD (post), non-fungal AECOPD after treatment; *Candida* (pre), *Candida*-positive AECOPD before antifungal treatment; *Candida* (post), *Candida*-positive AECOPD after antifungal treatment; and *Aspergillus* (pre), *Aspergillus*-associated AECOPD before antifungal treatment. “Pre” and “post” indicate samples collected before treatment initiation and at the end of the treatment course, respectively. Post-treatment samples were available for only two patients in the *Aspergillus*-associated group; therefore, no formal longitudinal pre/post-treatment comparison was performed for this group. Candidate differential metabolites were selected using a variable importance in projection (VIP) score ≥1 and a nominal two-sided Student’s *t*-test *P* value <0.05. No formal false discovery rate (FDR) correction was applied; therefore, metabolites meeting these criteria should be interpreted as exploratory candidate features rather than statistically confirmed differential metabolites.

**Figure 7:**
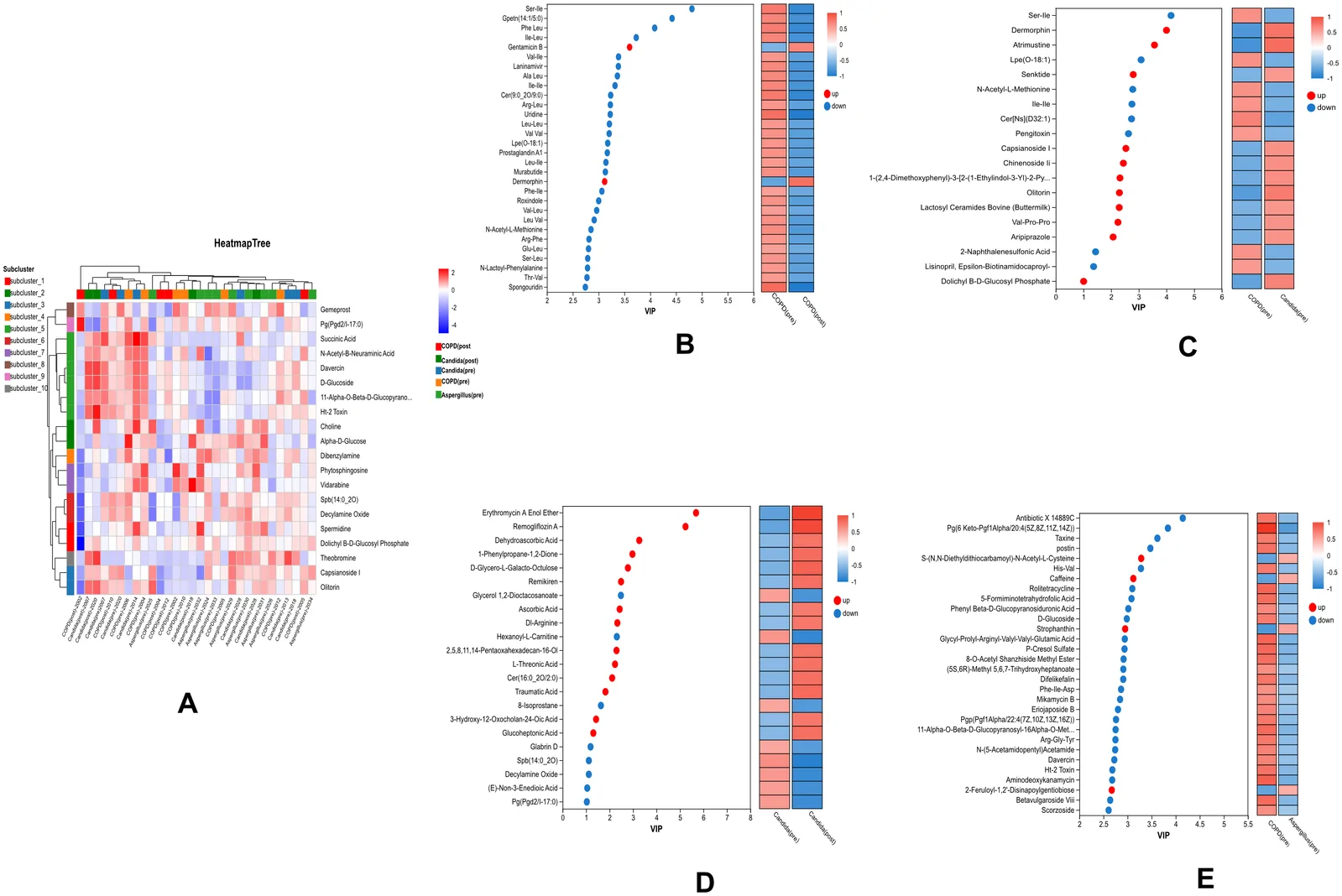
Key differential metabolite signatures across COPD subgroups. COPD (pre), non-fungal AECOPD before treatment; COPD (post), non-fungal AECOPD after treatment; Candida (pre), *Candida*-positive AECOPD before antifungal treatment; Candida (post), *Candida*-positive AECOPD after antifungal treatment; Aspergillus (pre), *Aspergillus*-associated AECOPD before antifungal treatment. Pre and post indicate samples collected before treatment initiation and at the end of the treatment course, respectively. Post-treatment samples were available for only two patients in the Aspergillus-associated group; therefore, no formal longitudinal pre/post-treatment comparative analysis was performed for this group. **(A)** Hierarchical clustering heatmap showing the abundance patterns of selected differential metabolites across the study samples. **(B)** VIP-ranked metabolites distinguishing COPD(post) from COPD(pre). **(C)** VIP-ranked metabolites distinguishing Candida(pre) from COPD(pre). **(D)** VIP-ranked metabolites distinguishing Candida(post) from Candida(pre). **(E)** VIP-ranked metabolites distinguishing Aspergillus(pre) from COPD(pre).

Pathway enrichment analysis of baseline comparisons suggested widespread metabolic differences associated with *Aspergillus* status, including sphingolipid metabolism, purine metabolism, one-carbon metabolism, and cAMP/cGMP signaling pathways. In secondary exploratory longitudinal comparisons, pre/post-treatment differences involved purine/pyrimidine and sulfur metabolism in the non-fungal group and glutathione and arachidonic acid metabolism in the *Candida*-positive group (**Figure 8**). These longitudinal findings were not evaluated in the *Aspergillus*-associated group because of insufficient paired post-treatment samples.

**Figure 8:**
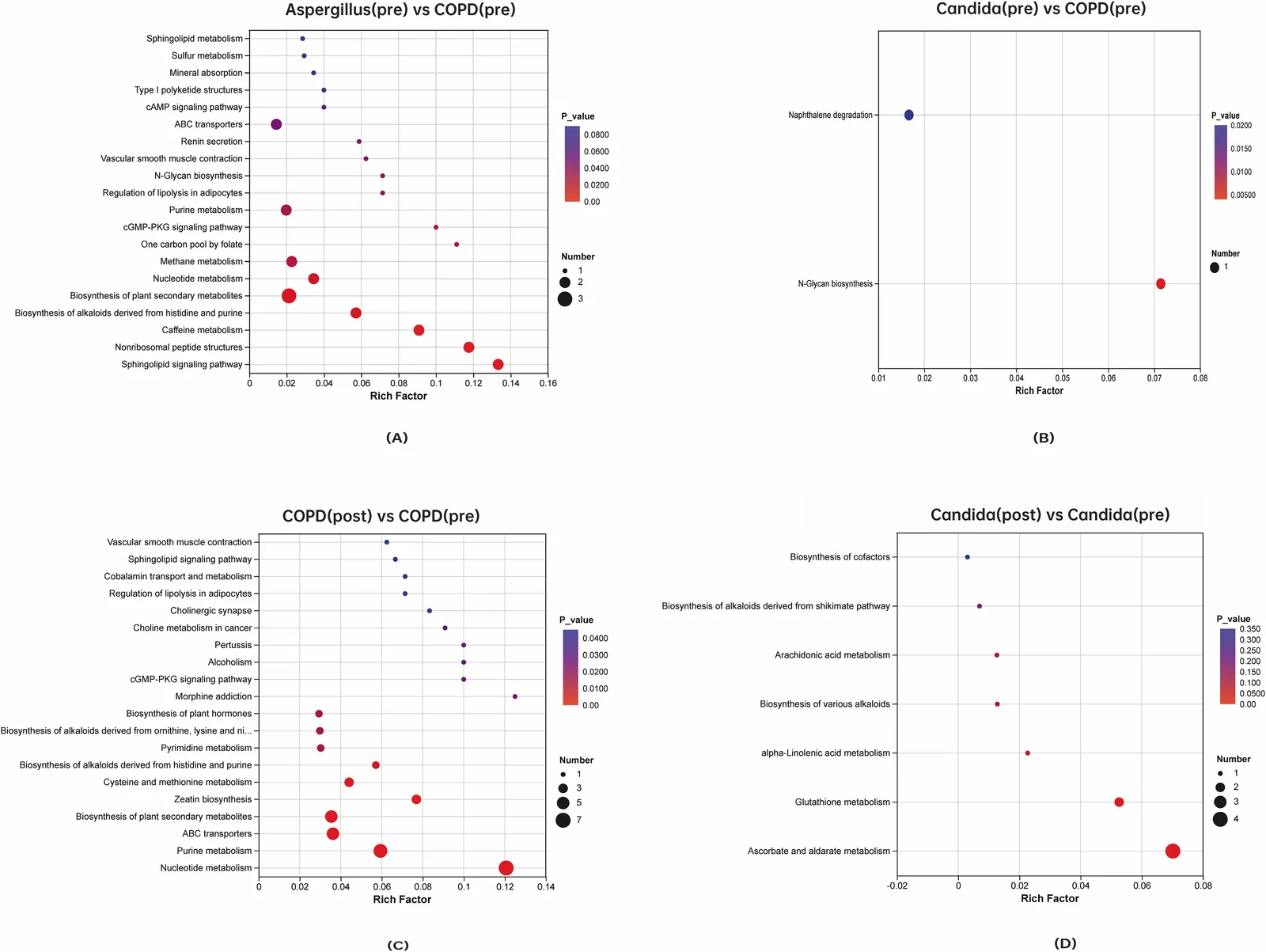
KEGG pathway enrichment of differential metabolites. COPD (pre), non-fungal AECOPD before treatment; COPD (post), non-fungal AECOPD after treatment; Candida(pre), *Candida*-positive AECOPD before antifungal treatment; Candida(post), *Candida*-positive AECOPD after antifungal treatment; Aspergillus(pre), *Aspergillus*-associated AECOPD before antifungal treatment. Pre and post indicate samples collected before treatment initiation and at the end of the treatment course, respectively. Post-treatment samples were available for only two patients in the Aspergillus-associated group; therefore, no formal longitudinal pre/post-treatment comparative analysis was performed for this group. Bubble color represents the pathway-enrichment P value, with lower P values shown in red and higher P values shown in blue/purple; bubble size represents the number of differential metabolites mapped to each pathway. Because the P-value ranges differ among comparisons, each panel is presented with its own numerical color scale. **(A)** *Aspergillus*(pre) vs COPD (pre). **(B)** *Candida* (pre) vs COPD (pre). **(C)** COPD (post) vs COPD (pre). **(D)** *Candida* (post) vs *Candida* (pre).

### Integrated microbiome-metabolome signatures of *Aspergillus*-associated AECOPD

Procrustes analysis did not support global concordance between microbial community structure and metabolomic profiles (M² = 0.797, P = 0.199). However, O2PLS identified a dominant shared latent component differentiating Aspergillus-associated AECOPD from non-fungal AECOPD (R2X = 0.612; R2Y = 0.476). Microbial features contributing to this shared component included *Veillonella*, *Porphyromonas*, and *Clostridia_UCG-014*, whereas contributing metabolite features included propionate, succinate, and pyruvate (**Figure 9**; **Supplementary Tables S5**, **S6**). These joint loading patterns represent feature contributions to the shared cross-omics covariance structure and should not be interpreted as direct pairwise associations between individual taxa and metabolites.

**Figure 9:**
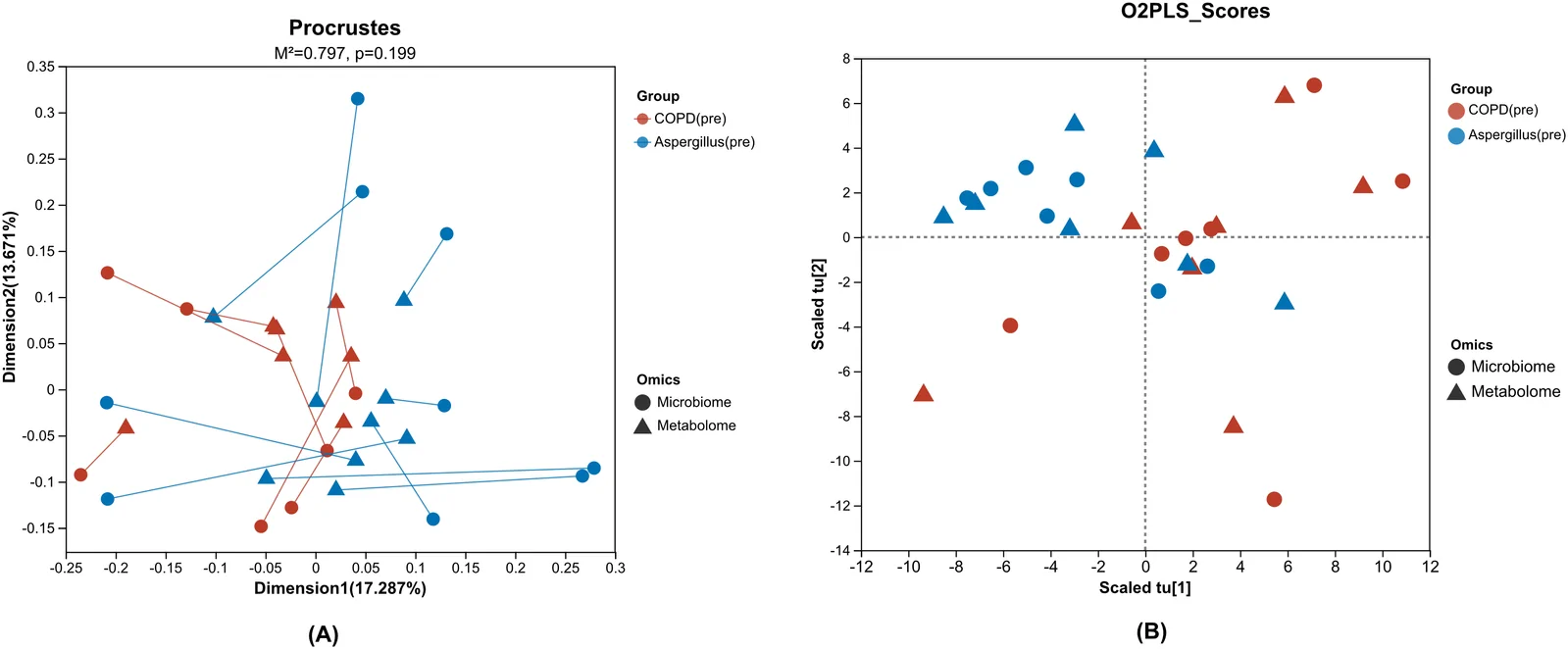
Integration analysis of oral microbiome and metabolomic profiles in COPD (pre) and Aspergillus(pre) groups. COPD (pre), non-fungal AECOPD before treatment; Aspergillus(pre), *Aspergillus*-associated AECOPD before antifungal treatment. “Pre” indicate samples collected before treatment initiation. **(A)** Procrustes analysis comparing microbiome and metabolome ordinations. **(B)** O2PLS score plot showing integrated variation between omics datasets.

## Discussion

This integrative multi-omics study identified distinct pretreatment oral microbial and metabolic profiles associated with fungal status in AECOPD. The primary findings were derived from cross-sectional pretreatment comparisons, in which the *Aspergillus*-associated group showed the most pronounced differences in microbial community structure and metabolic profiles relative to non-fungal AECOPD. Longitudinal pre/post-treatment analyses were secondary and exploratory and were restricted to the non-fungal and *Candida*-positive groups because only two post-treatment samples were available in the *Aspergillus*-associated group. Accordingly, our findings support a distinct baseline oral microbial-metabolic phenotype associated with *Aspergillus* status but do not establish the longitudinal treatment response of *Aspergillus*-associated AECOPD.

Pulmonary aspergillosis occurs within a complex respiratory microbial ecosystem, in which microbial community composition and the surrounding metabolic milieu may influence disease development and clinical outcomes (Cai et al., 2022; Rozaliyani et al., 2023). Previous studies have specifically reported alterations in the lower-airway microbiome among patients with invasive pulmonary aspergillosis. Herivaux et al. reported reduced bacterial alpha diversity and altered abundances of several taxa in bronchoalveolar lavage samples from patients with invasive pulmonary aspergillosis (Herivaux et al., 2022). Similarly, Ao et al. reported differences in lung microbial composition between patients with and without invasive pulmonary aspergillosis (Ao et al., 2023). Collectively, these studies examined the lower-airway rather than the oral microbiome and are cited solely to provide contextual evidence of altered microbial ecology associated with pulmonary aspergillosis. Because paired oral and lower-airway samples were not collected in our study, whether these lower-airway findings correspond to the observed oral microbial patterns remains unknown.

Regarding the *Candida*-positive group, *Candida albicans* is a common member of the human mucosal microbiota (Schille et al., 2025). Its commensal status may provide biological context for the comparatively modest oral microbial differences observed in this group. However, the present study cannot determine whether *Candida*-positive status itself contributed to these differences.

Importantly, the relative contribution of infectious pathogens should be interpreted within the specific clinical context. In an analysis of fever of unknown origin in China, Kang and Zheng reported that fungal infections accounted for 0.8% of cases during 2013–2022, compared with 10.0% for bloodstream infections and 9.3% for tuberculosis, illustrating marked differences in the proportional contribution of infectious etiologies across clinical settings (Kang and Zheng, 2024). However, that study did not distinguish between *Aspergillus* and *Candida* and was not conducted in patients with AECOPD; therefore, these proportions cannot be directly extrapolated to our population. Moreover, because our study used a balanced subgroup design, it was not intended to estimate or compare the prevalence or etiological contribution of *Aspergillus* and *Candida*. Rather, our findings suggest differences in their ecological and metabolic associations with AECOPD: the *Aspergillus*-associated group showed more pronounced baseline oral microbial dysbiosis and metabolic alterations than the *Candida*-positive group. These observations should therefore be interpreted as differences in microbial–metabolic phenotype rather than evidence that *Aspergillus* is more prevalent or etiologically more important than *Candida* in AECOPD. The observed differences may reflect their distinct biological properties, with *Aspergillus* having greater potential for invasive growth and immune activation, whereas *Candida* more commonly persists as a mucosal commensal.

PICRUSt2-based functional prediction showed broadly similar profiles across groups, with modest descriptive variation in pathways related to environmental sensing, membrane transport, and energy metabolism. Because these profiles were inferred from 16S rRNA gene data and were not subjected to formal between-group differential testing, they should be regarded as exploratory hypotheses rather than statistically supported functional alterations. Validation using shotgun metagenomics, metatranscriptomics, or targeted functional assays is required. Metabolomic profiling further identified candidate alterations involving sphingolipid signaling, nucleotide metabolism, and one-carbon metabolic pathways, which may participate in fungal–host metabolic and immune interactions (Li et al., 2026). Although global microbiome–metabolome concordance was not observed, O2PLS identified a shared cross-omics covariance structure to which subsets of microbial and metabolite features contributed. These joint loading patterns reflect feature contributions to a shared latent component rather than direct pairwise taxon–metabolite associations. Consistent with previous findings (Taubenheim et al., 2025), the observed metabolic shifts may reflect the combined influences of microbial compositional changes and host–microbe–inflammatory interactions. These findings suggest that the shared cross-omics signal may be concentrated within a limited subset of microbial and metabolite features rather than reflected by ecosystem-wide concordance. However, direct relationships among these features require dedicated pairwise association or network analyses.

Accumulating evidence supports the oral–lung axis as a biologically plausible link between oral microbial ecology and lower airway disease, with microaspiration and shared mucosal immune signaling representing potential mechanisms connecting these compartments (Garmendia and Cebollero-Rivas, 2024; Zhou et al., 2025; Zygmunt et al., 2025). In line with this framework, our findings identify distinct oral microbial–metabolic signatures associated with *Aspergillus*-associated AECOPD. Given the non-invasive and repeatable nature of oral sampling, these signatures may provide a useful basis for future biomarker development. However, paired sputum or bronchoalveolar lavage fluid (BALF) samples were not collected concurrently; therefore, the present study cannot determine whether the observed oral alterations are directly reflected in the lower airways or associated with local airway inflammation. Oral sampling should thus be regarded as a complementary compartment for exploratory profiling rather than as a validated surrogate for lower-airway pathology. Moreover, the clinical relevance of these signatures remains to be established, because the present study did not evaluate associations with clinical outcomes such as hospitalization duration, mechanical ventilation, readmission, or longitudinal lung-function decline. Accordingly, these findings should be regarded as candidate phenotype-associated signatures rather than clinically validated biomarkers.

Several limitations should be acknowledged. First, the sample size was relatively modest, particularly after subgroup stratification and pre/post-treatment comparisons, which may limit statistical power and generalizability. Although we applied an integrated clinical, microbiological, and radiological assessment to minimize the inclusion of simple fungal colonization, histopathological confirmation was unavailable in most patients. Therefore, residual misclassification between colonization and clinically relevant fungal infection cannot be completely excluded. In addition, patients who had received antibiotics within 4 weeks before enrollment were excluded to minimize the confounding effects of recent antibiotic exposure on oral microbial and metabolomic profiles. However, this criterion may have introduced selection bias and limited the representativeness of our cohort, as many patients with AECOPD receive antibiotics in real-world clinical practice. Second, although we focused on oral bacterial and metabolic alterations, we did not directly characterize the oral mycobiome, limiting our ability to delineate cross-kingdom interactions or determine the extent to which fungal burden contributed to the observed ecological shifts. Moreover, although *Aspergillus*-associated AECOPD showed the most pronounced oral microbial and metabolic alterations in our cohort, these changes are unlikely to be attributable to fungal status alone. The oral ecosystem is dynamically shaped by interactions among microorganisms, epithelial barriers, and local host immune responses. Local immunoregulatory pathways, including IDO-, TGF-β-, and ATP-related signaling, may contribute to remodeling of the oral inflammatory and ecological microenvironment (Chansaenroj et al., 2024). Because these host immune pathways were not directly measured, their contribution remains speculative and warrants further investigation. Third, paired lower-airway samples, such as sputum or BALF, were not collected concurrently with the oral samples. Although microaspiration provides a biologically plausible mechanism linking the oral and lower-airway microbiota, the present study cannot establish direct concordance between oral microbial-metabolic alterations and lower-airway microbial composition or local inflammatory activity. Accordingly, the oral signatures identified here should not be interpreted as validated surrogates of lower-airway pathology. Future studies incorporating paired oral and lower-airway sampling together with airway inflammatory measurements are required to directly evaluate the oral–lung relationship. Fourth, although the expanded Human Oral Microbiome Database (eHOMD) may provide improved species-level resolution for oral bacterial taxa, taxonomic annotation in this study was based on SILVA. Therefore, some oral taxa may have been classified at a lower taxonomic resolution. In addition, the LEfSe analysis used nominal Kruskal–Wallis P values together with an LDA effect-size threshold without formal FDR correction. Consequently, some identified taxa may represent false-positive findings, and the LEfSe-derived signatures should be considered exploratory until validated in larger independent cohorts using multiple-testing-adjusted differential abundance methods. Moreover, functional interpretation was based on PICRUSt2 predictions derived from 16S rRNA gene profiles rather than direct measurements by shotgun metagenomics or transcriptomics. Given the modest sample size and incomplete reference genome representation of many uncultivated oral microorganisms, the accuracy and generalizability of these predicted functional profiles are inherently limited. Therefore, pathway differences, including those involving quorum sensing, two-component systems, and membrane transport, should be regarded as exploratory and hypothesis-generating rather than as confirmed functional alterations. Validation using shotgun metagenomics, meta-transcriptomics, or targeted functional assays will be required in future studies. In addition, multiple metabolite-wise comparisons were performed using nominal Student’s t-test P values without formal FDR correction. Although candidate metabolites were jointly screened using VIP and nominal P-value criteria and the supervised models were evaluated using cross-validation and permutation testing, these procedures do not replace formal multiple-testing correction. Therefore, the identified metabolic features should be regarded as exploratory and require validation in larger independent cohorts. Fifth, post-treatment sample collection was incomplete in the *Aspergillus*-associated group, largely because of the prolonged antifungal treatment course and post-discharge follow-up constraints. Only two post-treatment samples were available; therefore, this group was excluded from formal longitudinal pre/post-treatment analyses. Accordingly, the primary conclusions of this study are based on cross-sectional pretreatment comparisons across fungal-status groups, whereas longitudinal findings in the non-fungal and *Candida*-positive groups should be regarded as secondary and exploratory. The present study therefore does not permit comparative assessment of treatment response across all three groups or conclusions regarding longitudinal treatment-associated changes in *Aspergillus*-associated AECOPD. Moreover, differences in concomitant antibiotic exposure and antifungal regimens may have influenced the observed longitudinal changes. Sixth, the present study was not designed or powered to evaluate the prognostic significance of the identified microbial and metabolic signatures. Clinical outcomes such as length of hospital stay, mechanical ventilation, readmission, and longitudinal lung-function decline were not systematically linked to the multi-omics features. Therefore, the clinical utility of these signatures for prognosis, risk stratification, or treatment monitoring remains uncertain. Larger prospective cohorts with standardized longitudinal follow-up and predefined clinical endpoints are required to determine their prognostic relevance. Finally, the largely cross-sectional design precludes causal inference. Future studies integrating multi-site sampling along the oral–airway axis, longitudinal follow-up, comprehensive immune phenotyping, and clinically relevant outcomes will be essential to clarify temporal dynamics, mechanistic links, and the clinical significance of oral ecological signatures in *Aspergillus*-associated AECOPD.

## Conclusion

In summary, *Aspergillus*-associated AECOPD was associated with pronounced pretreatment oral microbial dysbiosis and metabolic alterations, supporting the presence of a distinct oral microbial–metabolic phenotype. These exploratory candidate signatures support further investigation of the oral microenvironment as a clinically accessible compartment in AECOPD. Their prognostic utility requires validation in larger longitudinal cohorts with well-defined clinical outcomes, while paired oral and lower-airway studies are needed to determine whether they reliably reflect lower-airway microbial and inflammatory alterations.

## Data Availability

The datasets presented in this study can be found in online repositories. The names of the repository/repositories and accession number(s) can be found in the article/**Supplementary Material**.

## Supplementary material

Supplementary Figure 1 Relative standard deviation (RSD) distribution of metabolic features in pooled quality-control samples. The cumulative distribution of RSD values across pooled quality-control (QC) samples is shown for the raw data (Raw_QC) and the preprocessed data (Pre_QC). The vertical dashed line indicates an RSD threshold of 30%. At this threshold, 76.34% of metabolic features in the raw QC data and 86.84% of features in the preprocessed QC data had RSD values below 30%. Data preprocessing included missing-value filtering and recoding, normalization, QC validation, and data transformation.
