# Supplementary material for: Oral microbial and metabolic alterations in acute exacerbations of COPD across fungal status
Source: Front Cell Infect Microbiol. 2026 Oct 15;16:1843568. doi: 10.3389/fcimb.2026.1843568 (PMC13716569; doi:10.3389/fcimb.2026.1843568)
Supplement: Supplementary file 1 [file DataSheet1.pdf]

Table S1. Individual-level clinical, microbiological, radiological, and diagnostic characteristics of the study participants

Patient ID	Study group	Respiratory specimen culture	GM test	BDG test	NGS result	Chest CT findings	Host/clinical factors	Histopathology	Final diagnostic category
2006	Control	Negative	Not performed	Not performed	Not performed	Scattered patchy opacities in both lungs	AECOPD; diabetes mellitus	Not performed	AECOPD without evidence of fungal infection
2004	Control	Negative	Negative	Negative	Not performed	Linear opacity in the lingular segment of the left upper lobe, compatible with focal atelectatic change	AECOPD	Not performed	AECOPD without evidence of fungal infection
2005	Control	Negative	Not performed	Not performed	Not performed	Not performed	AECOPD; diabetes mellitus	Not performed	AECOPD without evidence of fungal infection

2002	Control	Negative	Negative	Negative	Not performed	Bilateral multilobar patchy opacities	AECOPD	Not performed	AECOPD without evidence of fungal infection
2012	Control	Sputum culture: Moraxella catarrhalis	Negative	Negative	Not performed	Left upper lobe apicoposterior patchy opacity with bilateral bronchial wall thickening and scattered peribronchial opacities	AECOPD; diabetes mellitus	Not performed	AECOPD without evidence of fungal infection
2010	Control	Sputum culture: Stenotrophomonas maltophilia	Negative	Negative	Not performed	Enlarged cardiac silhouette, dilated pulmonary arteries, and a small left pleural effusion	AECOPD; broad-spectrum antibiotic exposure	Not performed	AECOPD without evidence of fungal infection

2014	Candida	Sputum culture: Candida albicans	Negative	Negative	Not performed	Bilateral bronchial wall thickening with patchy opacities, bilateral bronchiectasis, and pulmonary nodules	AECOPD; persistent or worsening respiratory symptoms despite antibacterial therapy; broad-spectrum antibiotic exposure	Not performed	Probable Candida co-infection

2013	Candida	Sputum culture: Candida albicans	Negative	Negative	Not performed	Bilateral interstitial pulmonary edema with cardiomegaly, small bilateral pleural effusions slightly increased from prior imaging, and bilateral patchy inflammatory opacities with pulmonary nodules	AECOPD; persistent or worsening respiratory symptoms despite antibacterial therapy; broad-spectrum antibiotic exposure	Not performed	Probable Candida co-infection

2020	Candida	Sputum culture: Candida albicans and Acinetobacter baumannii	Negative	Negative	Not performed	Patchy, nodular, and linear opacities in both upper lobes and the dorsal segments of both lower lobes	AECOPD; ICU admission; persistent or worsening respiratory symptoms despite antibacterial therapy; broad-spectrum antibiotic exposure	Not performed	Probable Candida co-infection
2026	Candida	Sputum culture: Candida albicans and Pseudomonas aeruginosa	Negative	Negative	Not performed	Bronchiectasis with inflammatory changes and a left pulmonary nodule	AECOPD; ICU admission; worsening respiratory symptoms; broad-spectrum antibiotic exposure	Not performed	Probable Candida co-infection

2018	Candida	Sputum culture: Candida spp., Pseudomonas aeruginosa (outside hospital), and Haemophilus influenzae	Negative	Negative	Not performed	Not performed	AECOPD; diabetes mellitus; persistent or worsening respiratory symptoms despite antibacterial therapy; broad-spectrum antibiotic exposure	Not performed	Probable Candida co-infection
2007	Candida	BALF culture: Pseudomonas aeruginosa, Candida glabrata, and Candida albicans	Negative	Negative	Not performed	Bilateral bronchiectasis with surrounding patchy infectious opacities	AECOPD; persistent or worsening respiratory symptoms despite antibacterial therapy; broad-spectrum antibiotic exposure	Not performed	Probable Candida co-infection

2024	Aspergillus	Sputum culture: Escherichia coli and Aspergillus fumigatus	Negative	Negative	Not performed	Mild inflammatory changes in the left lower lobe with bilateral pulmonary nodules	AECOPD; persistent or worsening respiratory symptoms despite antibacterial therapy; broad-spectrum antibiotic exposure	Not performed	Probable Aspergillus co-infection
2032	Aspergillus	Sputum culture: Aspergillus fumigatus	Negative	Negative	mNGS: Aspergillus fumigatus	Bilateral bronchiectasis with infectious changes	AECOPD; persistent or worsening respiratory symptoms despite antibacterial therapy; broad-spectrum antibiotic exposure	Not performed	Probable Aspergillus co-infection

2029	Aspergillus	Sputum culture: Aspergillus fumigatus complex and Aspergillus flavus/oryzae	1.32	233.11	tNGS: Aspergillus fumigatus complex, Aspergillus flavus/oryzae, and Corynebacterium striatum	Bilateral inflammatory opacities with bronchiectasis	AECOPD; diabetes mellitus; persistent or worsening respiratory symptoms despite antibacterial therapy; broad-spectrum antibiotic exposure	Not performed	Probable Aspergillus co-infection

2030	Aspergillus	Sputum culture: Aspergillus fumigatus complex	Negative	Negative	Not performed	Multifocal bilateral infectious opacities with areas of consolidation	AECOPD; diabetes mellitus; persistent or worsening respiratory symptoms despite antibacterial therapy; broad-spectrum antibiotic exposure	Not performed	Probable Aspergillus co-infection
2028	Aspergillus	Sputum culture: Aspergillus fumigatus and Aspergillus flavus	0.21	156.95	Not performed	Bronchitis with peribronchial inflammatory changes and pulmonary nodules	AECOPD; persistent or worsening respiratory symptoms despite antibacterial therapy; broad-spectrum antibiotic exposure	Not performed	Probable Aspergillus co-infection

2031	Aspergillus	Sputum culture: Aspergillus fumigatus, Pseudomonas aeruginosa, and Moraxella catarrhalis	Negative	Negative	Not performed	Multifocal bilateral infectious opacities with areas of consolidation and cavitation, bilateral bronchiectasis, pleural effusion, and pericardial effusion	AECOPD; worsening respiratory symptoms; broad-spectrum antibiotic exposure	Not performed	Probable Aspergillus co-infection

Abbreviations: AECOPD, acute exacerbation of chronic obstructive pulmonary disease; BALF, bronchoalveolar lavage fluid; GM, galactomannan; BDG, (1→3)-β-D-glucan; NGS, next-generation sequencing; mNGS, metagenomic NGS; tNGS, targeted NGS; ICU, intensive care unit.

Table S2. Treatment exposures and sampling schedule among non-fungal, Candida-positive, and Aspergillus-associated AECOPD patients

Group	Non-fungal AECOPD (n=6)	*Candida*-positive AECOPD (n=6)	*Aspergillus*-associated AECOPD (n=6)	P value
Antibiotic therapy, n (%)	5 (83.3%)	6 (100.0%)	6 (100.0%)	—
Antibiotic class, n (%)				
β-lactam/β-lactamase inhibitor	5 (83.3%)	5 (83.3%)	6 (100.0%)	—
Cephalosporin-based	1 (16.7%)	1 (16.7%)	2 (33.3%)	—
Carbapenem	0 (0%)	3 (50.0%)	1 (16.7%)	—
Fluoroquinolone	0 (0%)	0 (0%)	1 (16.7%)	—
Antibiotic duration, d*	7.3 ± 4.8	6.7 ± 3.3	12.3 ± 2.6	0.034
Cumulative antibiotic exposure, antibiotic-days†	7.3 ± 4.8	8.8 ± 3.7	16.8 ± 9.0	0.04
Antifungal agent	none	Caspofungin/Fluconazole	Voriconazole	
antifungal Duration (d)	0	12.5 ± 1.9	52.5 ± 4.9	
ICS type	Budesonide	Budesonide	Budesonide	
ICS daily dose(mg/d)	4	4	4	-
ICS duration(d)	8.9 ± 1.1	8.3 ± 1.6	8.3 ± 2.2	0.781
Cumulative ICS exposure, mg·d	35.6 ± 4.4	33.2 ± 6.4	33.2 ± 8.8	0.781
Antifungal therapy, n (%)	0 (0%)	4 (66.7%)	6 (100.0%)	—

Antifungal agent	None	Caspofungin (4/6); Fluconazole (2/6)	Voriconazole (6/6); +caspofungin (2/6); +isavuconazole (1/6)	—
Cumulative antifungal exposure, antifungal-days‡	0	9.3 ± 3.6	29.5 ± 2.1(n=2)	—
Pre-treatment sampling	Before treatment initiation(n=6)	Before treatment initiation(n=6)	Before treatment initiation(n=6)	
Post-treatment sampling	After standard AECOPD treatment(n=6)	At the end of antifungal therapy(n=4)	At the end of antifungal therapy(n=2)	

Notes

* Antibiotic duration was defined as the longest recorded single-antibiotic course for each patient; patients without antibiotics were counted as 0 d.

† Cumulative antibiotic exposure was calculated as the sum of recorded antibiotic-specific treatment days (antibiotic-days). Daily doses were not available and heterogeneous agents should not be combined as mg·d.

‡ For multi-agent antifungal regimens, this is the sum of recorded agent-specific treatment days (drug-days), not necessarily elapsed calendar duration if treatments overlapped.

P values for antibiotic duration/exposure are from one-way ANOVA using the de-identified derived values; the ICS P value was calculated from the provided group summary statistics.

ICS rows and sampling schedule were retained from the user's supplied summary because corresponding patient-level hospitalization ICS/sampling fields were not identifiable in the uploaded raw sheet.

Table S3 Subject Characteristics for Subjects with Evaluable Oral Wash

	Non-fungal AECOPD (6)	*Candida*-positive AECOPD (6)	Aspergillus-associated AECOPD (6)	P-Value
Demographics				
Sex, Male(%)	16(100.0)	8(88.9)	8(88.9)	0.389
Age, mean(SD)	72.44(7.55)	75.33(7.79)	70.89(6.07)	0.426
BMI, mean(SD)	21.45(3.03)	22.07(2.91)	20.90(5.11)	0.796
Clinical History				
Years with COPD	8.69(5.64)	11.56(3.28)	15.33(14.23)	0.179
Diabetes Mellitus(%)	4(25.0)	3(33.3)	2(22.2)	0.853
Hypertension(%)	6(37.5)	3(33.3)	4(44.4)	0.886
Cardiovascular Disease(%)	3(18.8)	3(33.3)	1(11.1)	0.491
Inhaled corticosteroid use(%)	6(37.5)	7(77.8)	2(22.2)	0.046
mMRC	2.4(1.2)	1.9(1.1)	2.4(1.4)	0.45
Pack-years of smoking, mean(SD)	427.56(250.40)	365.00(182.50)	223.44(177.26)	0.094
Current tobacco use, Yes(%)	5(31.2)	3(33.3)	5(55.6)	0.457
Current alcohol use, Yes(%)	13(81.2)	9(100.0)	9(100.0)	0.157
Symptoms at Presentation				
Fever	1(6.2)	3(33.3)	0(0.0)	0.058

Cough	16(100.0)	9(100.0)	9(100.0)	1
Sputum production	16(100.0)	9(100.0)	9(100.0)	1
Dyspnea	16(100.0)	9(100.0)	7(77.8)	0.052
Hemoptysis	0(0.0)	0(0.0)	1(11.1)	0.239
Laboratory Parameters				
White Blood Cell Count(x10^9/L)	7.74(3.51)	7.46(2.66)	6.84(1.75)	0.761
Neutrophil Count	6.03(3.46)	6.16(2.64)	5.42(1.90)	0.840
Lymphocyte Count	1.02(0.55)	0.68(0.50)	0.82(0.44)	0.267
CRP(mg/L)	34.81(31.03)	21.78(23.29)	49.69(49.76)	0.259
IL-6(pg/mL)	52.50(78.16)	64.32(82.33)	71.34(77.78)	0.839
Blood Gas Analysis				
$PaCO_2$(mmHg)	52.06(20.20)	69.78(26.33)	49.44(13.43)	0.078
PaO_2/FiO_2	298.81(101.32)	310.22(101.68)	292.44(94.74)	0.929

COPD, Chronic Obstructive Pulmonary Disease; BMI, Body Mass Index; CRP, c-reactive protein.

Table S4. Alpha diversity indices and pairwise comparisons among oral samples

	COPD (post)	COPD (pre)	Candida (post)	Candida (pre)	Aspergillus (pre)	P-value			
						COPD(post) vs COPD (pre)	Candida(post) vs Candida(pre)	Candida(pre) vs COPD (pre)	Aspergillus(pre) vs COPD (pre)
**Sobs**	44.40 ± 11.37	76.67 ± 20.76	26.00 ± 5.48	57.83 ± 23.16	42.67 ± 13.08	0.0173	0.0667	0.3095	0.012
**ACE**	44.40 ± 11.37	76.67 ± 20.76	26.08 ± 5.61	57.88 ± 23.10	42.75 ± 13.19	0.0173	0.0667	0.3095	0.012
**Chao**	44.40 ± 11.37	76.67 ± 20.76	26.00 ± 5.48	57.83 ± 23.16	42.67 ± 13.08	0.0173	0.0667	0.3095	0.012
**Shannon**	2.32 ± 0.28	2.99 ± 0.30	1.46 ± 0.34	2.55 ± 0.65	2.12 ± 0.35	0.0173	0.0667	0.5887	0.0016
**Simpson**	0.14 ± 0.04	0.09 ± 0.02	0.38 ± 0.15	0.15 ± 0.11	0.19 ± 0.06	0.0519	0.0381	0.5887	0.0076
**Coverage**	1.00000 ± 0.00000	1.00000 ± 0.00000	0.99999 ± 0.00002	0.99999 ± 0.00001	0.99999 ± 0.00002	1	0.8783	0.4047	0.0172

Data are presented as mean ± SD. Independent pretreatment groups were compared using the Wilcoxon rank-sum test. Within-group pre/post-treatment comparisons were performed using the Wilcoxon signed-rank test and included only participants with paired samples (non-fungal AECOPD, n=6 pairs; *Candida*-positive AECOPD,

n=4 pairs). No formal longitudinal comparison was performed in the *Aspergillus*-associated group because only two paired samples were available. P values <0.05 are shown in bold.

Table S5. O2PLS joint component loadings (pq[1]–pq[2]) for microbiome and metabolome features.

Objects	pq[1]	pq[2]	Omics
Phe-Leu-Ile	0.04895229	-0.0366007	metabolome
2-Hydroxypalmitic Acid	0.03563214	-0.0500952	metabolome
Gluconolactone	0.0531819	0.0335357	metabolome
Pe(P-16:0/0:0)	0.03290748	-0.0539255	metabolome
Pyruvic Acid	0.04207265	0.04601117	metabolome
2-Hydroxypentanoic Acid	0.04025911	0.0478275	metabolome
Subaphylline	0.03215393	-0.0561518	metabolome
Val-Leu	0.03766738	-0.050846	metabolome
6-Hydroxy-5-Methoxyindole Glucuronide	0.04893396	0.03981263	metabolome
4-Hydroxybutanoic Acid	0.04039984	0.04853603	metabolome
Lactic Acid	0.03815909	0.05087484	metabolome
4-Hydroxytamoxifen-O-Glucuronide	0.04427248	0.0447759	metabolome
Pg(Pgf1Alpha/I-21:0)	0.0510564	-0.0384116	metabolome
Ochromycinone	0.03575473	-0.0537943	metabolome
3-Ethoxypropanoic Acid	0.05272478	0.03692373	metabolome
7-Hydroxyoct-6-Enoylglycine	0.05177776	0.03797898	metabolome
Pc(18:1/0:0)	0.02089007	-0.0695625	metabolome
Entacapone	0.01463698	0.07604984	metabolome
7-Methylinosine	0.04414632	0.04658039	metabolome
Glutaconic Acid	0.03669722	0.05414448	metabolome
D-Alpha-Hydroxyglutaric Acid	0.03644033	0.05532365	metabolome
Teneligliptin	0.04574652	-0.0463935	metabolome
Propionic Acid	0.04787098	0.04523519	metabolome

Leu-Ser	0.02886044	-0.0664095	metabolome
Succinic Acid	0.04714522	0.04823975	metabolome
(R)-2-Hydroxystearic Acid	0.03954669	-0.0558737	metabolome
Pe(18:0/0:0)	0.04660093	-0.049661	metabolome
Lpe(O-18:1)	0.05237747	-0.0444416	metabolome
(E)-Non-3-Enedioic Acid	0.04854825	-0.049845	metabolome
D-Fucose	0.03945929	0.05961887	metabolome
unclassified_f__Atopobiaceae	0.06590494	0.11831985	microbiome
unclassified_f__Peptostreptococcaceae	0.06590494	0.11831985	microbiome
Litchfieldia	0.06590494	0.11831985	microbiome
unclassified_f__Lactobacillaceae	0.06590494	0.11831985	microbiome
Butyrivibrio	0.06590494	0.11831985	microbiome
Peptococcus	0.06590494	0.11831985	microbiome
Lactobacillus	0.06590494	0.11831985	microbiome
Prevotellaceae_UCG-004	0.03663273	-0.1506595	microbiome
Family_XIII_AD3011_group	0.03663273	-0.1506595	microbiome
Sphaerochaeta	0.03663273	-0.1506595	microbiome
Howardella	0.03663273	-0.1506595	microbiome
Pelospora	0.03663273	-0.1506595	microbiome
norank_f__Lachnospiraceae	0.0891239	0.09868147	microbiome
Hornefia	0.01453505	-0.1744486	microbiome
W5053	-0.0016165	-0.1900733	microbiome
norank_o__Clostridia_UCG-014	0.11725194	0.07777286	microbiome
norank_f__Selenomonadaceae	0.06289538	0.1333058	microbiome
Candidatus_Tammella	0.04216705	-0.1543229	microbiome

Catonella	0.10035271	0.09716495	microbiome
unclassified_f__Saccharimonadaceae	0.10955622	0.08923501	microbiome
[Eubacterium]_saphenum_group	-0.0019644	-0.1976086	microbiome
unclassified_f__Actinomycetaceae	0.10372433	-0.0991027	microbiome
Mogibacterium	0.11122606	-0.0943031	microbiome
unclassified_o__Lactobacillales	0.10299499	0.11251258	microbiome
unclassified_c__Actinobacteria	0.12619516	0.09358444	microbiome
Porphyromonas	0.10629273	-0.1161173	microbiome
Parascardovia	0.11175108	0.11230847	microbiome
Veillonella	-0.1137394	0.11338379	microbiome
norank_f__P5D1-392	0.08675372	0.15034918	microbiome
Limosilactobacillus	0.11097938	0.13326804	microbiome

pq[1] and pq[2] are variable loadings on the first and second joint O2PLS components. Variables with larger absolute loading values (|pq|) contribute more strongly to the cross-block covariation pattern between the untargeted metabolome and oral microbiome genus abundance. The sign of the loading indicates direction along the component (features with the same sign tend to vary concordantly, whereas opposite signs indicate inverse variation along that axis).

Table S6 O2PLS model performance and variance decomposition (R2X, R2Y, joint and orthogonal fractions)

R2X	R2Y	R2Xjoint	R2Yjoint	R2Xhat	R2Yhat	R2Xpred	R2Ypred
0.6119	0.4755	0.3771	0.4755	0.3235	0.4054	0.8578	0.8526

R2X and R2Y: the proportion of variance explained in the X-block (untargeted metabolome) and Y-block (oral microbiome genus abundance), respectively; R2X_joint and R2Y_joint: the shared (covarying) variation captured between X and Y; R2X_hat, R2Y_hat: data-specific variation in each block that is unrelated to the other block; R2X_pred and R2Y_pred: predictive explained variance estimated by cross-validation. O2PLS loadings indicate the contribution of each feature to the shared latent component. Features appearing within the same component should not be interpreted as having a direct pairwise association.

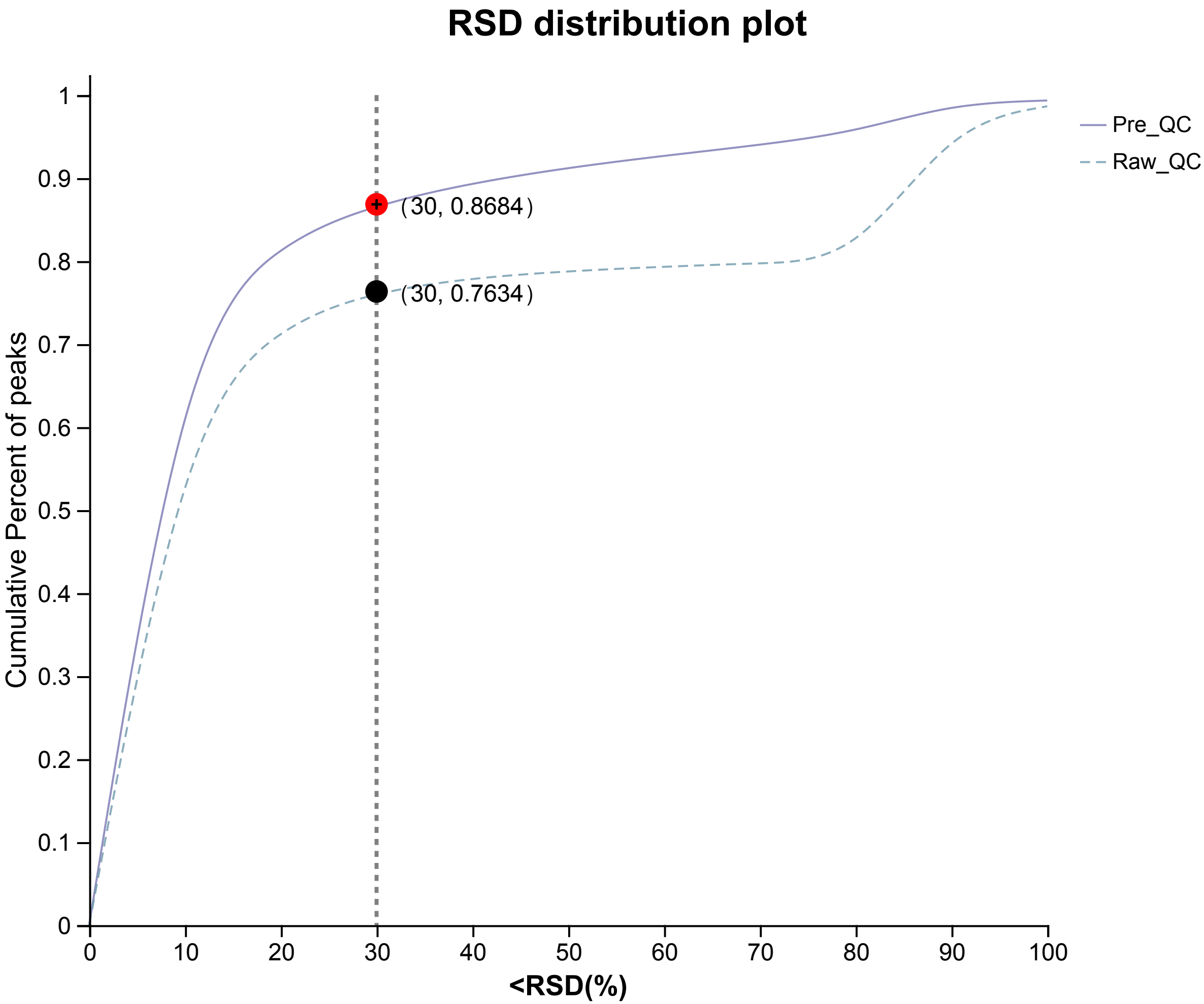
RSD distribution plot
Cumulative Percent of peaks
<RSD(%)
Pre_QC
Raw_QC
(30, 0.8684)
(30, 0.7634)
0
0.1
0.2
0.3
0.4
0.5
0.6
0.7
0.8
0.9
1
0
10
20
30
40
50
60
70
80
90
100
